# A novel approach for dose painting radiotherapy of brain metastases guided by mr perfusion images

**DOI:** 10.3389/fonc.2022.828312

**Published:** 2022-12-01

**Authors:** Chuanke Hou, Hanjing Yin, Guanzhong Gong, Lizhen Wang, Ya Su, Jie Lu, Yong Yin

**Affiliations:** ^1^ Department of Radiology, Beijing YouAn Hospital, Capital Medical University, Beijing, China; ^2^ Department of Radiation Physics, Shandong Cancer Hospital and Institute, Shandong First Medical University and Shandong Academy of Medical Sciences, Ji'nan, China

**Keywords:** brain metastases, radiotherapy, dose painting, 3D-arterial spin labeling, subvolume

## Abstract

**Purpose:**

To investigate the feasibility and dosimetric index features of dose painting guided by perfusion heterogeneity for brain metastasis (BMs) patients.

**Methods:**

A total of 50 patients with single BMs were selected for this study. CT and MR simulation images were obtained, including contrast-enhanced T1-weighted images (T1WI+C) and cerebral blood flow (CBF) maps from 3D-arterial spin labeling (ASL). The gross tumor volume (GTV) was determined by fusion of CT and T1WI+C images. Hypoperfused subvolumes (GTV_H_) with less than 25% of the maximum CBF value were defined as the dose escalation region. The planning target volume (PTV) and PTV_H_ were calculated from GTV and GTV_H_ respectively. The PTV_N_ was obtained by subtracting PTV_H_ from PTV, and conventional dose was given. Three kinds of radiotherapy plans were designed based on the CBF values. Plan 1 was defined as the conventional plan with an arbitrary prescription dose of 60 Gy for PTV. For dose painting, Plan 2 and Plan 3 escalated the prescription dose for PTV_H_ to 72 Gy based on Plan 1, but Plan 3 removed the maximum dose constraint. Dosimetric indices were compared among the three plans.

**Results:**

The mean GTV volume was 34.5 (8.4-118.0) cm^3^, and mean GTV_H_ volume was 17.0 (4.5-58.3) cm^3^, accounting for 49.3% of GTV. Both conventional plan and dose painting plans achieved 98% target coverage. The conformity index of PTV_H_ were 0.44 (Plan1), 0.64 and 0.72 (Plan 2 and Plan 3, *P<*0.05). Compared to Plan 1, the D_2%_, D_98%_ and D_mean_ values of the PTV_H_ escalated by 20.50%, 19.32%, and 19.60% in Plan 2 and by 24.88%, 17.22% and 19.22% in Plan 3 respectively (*P<*0.05). In the three plans, the index of achievement value for PTV_H_ was between 1.01 and 1.03 (*P<*0.05). The dose increment rates of Plan 2 and Plan 3 for each organs at risk (OARs) was controlled at 2.19% - 5.61% compared with Plan 1. The doses received by OARs did not significantly differ among the three plans (*P >*0.05).

**Conclusions:**

BMs are associated with significant heterogeneity, and effective escalation of the dose delivered to target subvolumes can be achieved with dose painting guided by 3D-ASL without extra doses to OARs.

## Introduction

Brain metastases (BMs) are the most common intracranial malignant tumors, and approximately 8-10% of tumor patients will develop BMs during their disease course (1). Approximately 30-50% of BMs patients die of uncontrolled and recurrent intracranial lesions (2). And large-volume BMs are highly heterogeneous due to their long growth cycle and complex blood supply (3, 4). Subvolumes with low cerebral blood flow (CBF) are hypoxic or potentially hypoxic areas, which are associated with radiation resistance (3). Ling et al. has proved that the tumor volume must be considered heterogeneous when assessing function and treatment response (5).

At present, radiotherapy (RT) is considered a curative-intent treatment for BMs patients primarily consisting of whole-brain radiotherapy (WBRT) and stereotactic radiosurgery (SRS) (6, 7). WBRT is suitable for multiple BMs and high-dose SRS plays an important role in treatment of small-volume BMs while it is limited for large-volume BMs (8). RT failure is usually manifested by insufficient local radiation doses and radiation-induced brain injury.

3D-arterial spin labeling (3D-ASL) perfusion imaging analyzes CBF parameters noninvasively and quantitatively and is independent of blood-brain barrier (BBB) damage (9). In addition, 3D-ASL has been widely used in clinical diagnosis, differential diagnosis and efficacy evaluation of brain tumors (9–11). Due to the tumor-specific characteristics of large-volume BMs, 3D-ASL is potentially promising for image-guided RT treatment planning with dose painting (12). Dose painting refers to the distribution of nonuniform radiation doses to target volumes according to functional or molecular images (13).

Increasing the doses to BMs can significantly improve the curative effect. Therefore, safe dose escalation for target subvolumes of BMs is crucial. This study therefore explored the feasibility of 3D-ASL-guided subvolume segmentation of BMs based on CBF map variation. In addition, we further studied the dosimetric indices of dose painting plans to provide additional reference for the formulation and modification of individualized RT to BMs patients.

## Materials and methods

### Patients

Fifty patients, namely, 29 males and 21 females (aged 33 - 74 years, with a median age of 57 years), with a single BMs who received RT were selected from July 2018 to September 2020. There were 33 cases of lung cancer, 9 of breast cancer, 4 of renal carcinoma, 2 of esophageal cancer and 2 of colon cancer. All patients were diagnosed with BMs with imaging and the maximum tumor cross-sectional diameter was more than 2 cm. The retrospective analysis of the medical records was approved by the Institutional Review Board of Shandong Cancer Hospital.

### Computed Tomography and MR Simulation

CT simulation images (slice thickness = 3 mm; slice gap = 3 mm) were obtained by Brilliance Big Bore CT scanner (Philips, Netherlands). MR imaging was performed on 3.0 T Discovery 750 W MR scanner (GE Healthcare, USA) with the same head position as CT simulation. 3D-ASL and contrast-enhanced T1-weighted (T1WI+C) images were acquired using 3D volume scanning (field of view, FOV= 26 cm; matrix size = 256×256; slice thickness = 3 mm). For 3D-ASL images, the special acquisition conditions were as follows: repetition time (TR) = 5160ms; echo time (TE) = 11.5 ms; and postlabeling delay (PLD) = 2025 ms. David et al. pointed out that it is more reasonable to set the PLD of healthy people older than 70 years old and adult clinical patients to around 2000ms (14). For T1WI+C images, TR = 8.5 ms; and TE = 3.2 ms. Gadopentetate dimeglumine was power-injected applying doses standardized by 0.2 mL/kg at 2 mL/s, and scan started 3 - 5 min after injection.

### Target volume definition

**Figure 1:**
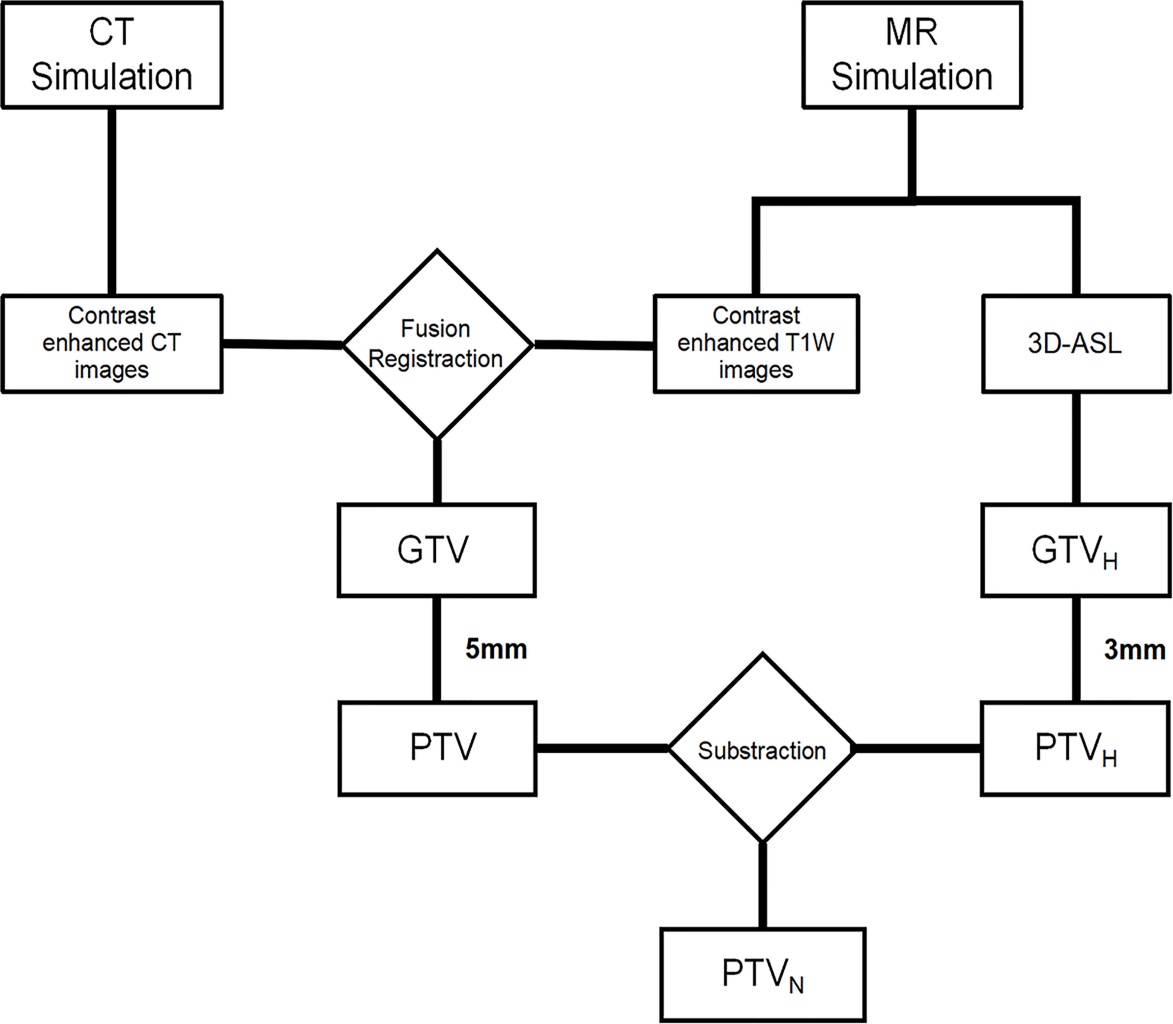
Target volume determination flow chart.

**Figure 2:**
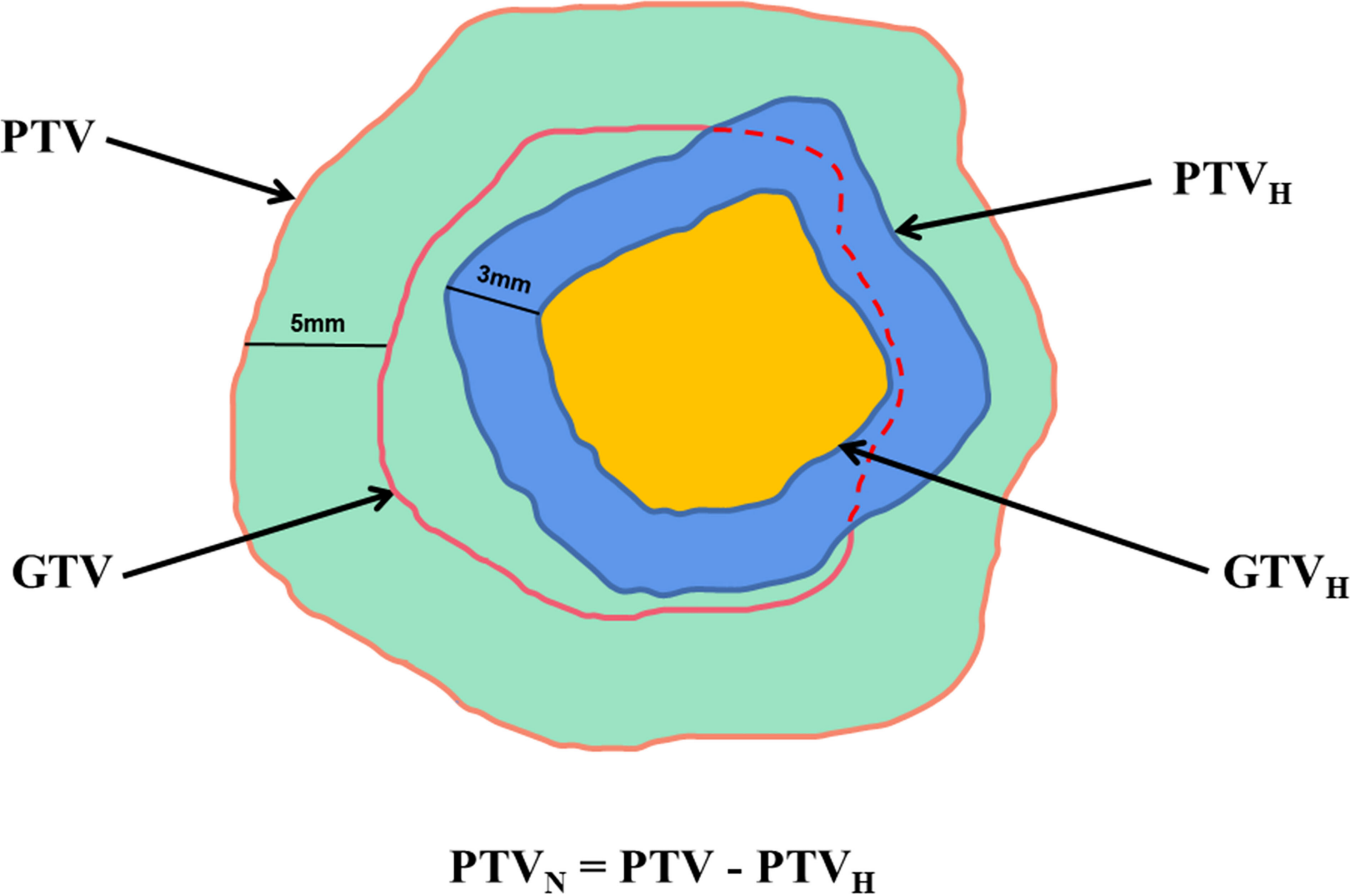
Illustration of the target volumes.

CT and T1WI+C images were fused in MIM Maestro software (6.8.8, USA), and gross tumor volume (GTV) was defined as the region with high signal. The GTV was divided into hypoperfused (GTV_H_), hyperperfused and nonperfused subvolumes based on CBF values (15). One-quarter of maximum CBF value was the junction of hypoperfused and hyperperfused subvolumes (16). Planning target volume (PTV) was designated as GTV plus a 5 mm margin, and 3 mm margins were added to the GTV_H_ to obtain PTV_H_. Then, the PTV_N_ was obtained by subtracting the PTV_H_ from the PTV, which was given normal prescription dose. Schematic diagrams are shown in **Figures 1**, **2**.

### Treatment plans

Conventional and dose painting plans were implemented by intensity-modulated radiotherapy (IMRT) and simultaneous integrated boost (SIB) IMRT. These plans were designed using Eclipse (Version 15.6, Varian, USA). For larger tumors, with the most common doses being 27 Gy in 3 fractions and 30 Gy in 5 fractions applying with SRS based on NCCN (17). Preliminary results from a randomized phase II trial comparing dose-intensification with standard-dose IMRT for newly diagnosed glioblastoma demonstrate that a higher radiation dose improves overall survival (18). The tolerance dose for brain to a single course of RT is 60 Gy in 2 Gy daily fractions (19). And both the hypoxic areas of BMs have the same characteristics of RT resistance with glioblastoma, so we refer to the treatment plan of glioblastoma (20). Here, we set the prescription dose at 60 Gy and the elevated dose at 72 Gy (20). Boosting tumor subvolume may increase tumor control probability (TCP), and a moderate boost dose (120% -150%) to hypoxic areas is also beneficial to increase TCP (21, 22). The conventional plan (60 Gy) and the dose painting plan (72 Gy) were both set at 2 Gy/d in this study. The conventional plan, designated Plan 1, prescribed 60 Gy to PTV with a maximum dose (D_max_) constraint of 66 Gy (110% of prescription dose). For dose painting, Plan 2 escalated the PTV_H_ to 72 Gy with a D_max_ constraint of 79 Gy (110% of prescription dose) based on Plan 1. Moreover, Plan 3 was designed depending on Plan 2 without D_max_ constraint.

The optimized parameters and dose constraints for organs at risk (OARs) among three plans were unified. OARs were restricted to the following doses: D_max_<50 Gy for eye, D_max_<54 Gy for optic nerve, D_max<_ 8 Gy for lens, and D_max_<54 Gy for brainstem.

Dose calculation was performed in anisotropic analytical algorithm optimization mode (version 15.512) using 6 MV X-rays. The calculated grid was 2.5 mm×2.5 mm, and the prescription dose was acquired to cover 95% of the target volume.

### Evaluation of plan dosimetric indices

For PTVs, the D_2%_, D_98%_ and D_mean_ (doses to 2%, 98% and 50% volume of the PTV, respectively) were the maximum, minimum and mean dose, respectively. The D_max_ values of eyeballs, optic nerves, lenses and brainstem were compared. Furthermore, the target coverage, conformity index (CI) and index of achievement (IOA) were calculated. The proposed IOA is formulated as the volume-weighted average of the deviation between prescription dose and planned dose (23).

The formulas for calculating CI and IOA are as follows:


$$\text{CI}=\frac{V_{t,ref}}{V_{t}}\times \frac{V_{t,ref}}{V_{ref}};\;IOA=1+\sqrt{\sum_{\text{K}=1}^{\text{K}}\sum_{\text{j}=1}^{\text{j}}\left[{\left(\frac{{\text{D}}_{\text{j}}-{\text{D}}_{\text{K},\text{RX}}}{{\text{D}}_{\text{K},\text{RX}}}\right)}^{2}\times \frac{{\text{dDVH}}_{\text{PTV}\left(\text{K}\right)}\left({\text{D}}_{\text{j}}\right)}{{\text{V}}_{\text{PTV}\left(\text{K}\right)}}\right]}$$

where *V_t_* represents the PTVs, *V_t,ref_* represents the target volume covered by prescription dose, and *V_ref_* represents the volume covered by prescription dose. where K is the total number of PTVs, D_K,RX_ is the prescription dose for the Kth PTV, and V_PTV(K)_ is the volume of the kth PTV, where j is the total number of volumes, D_j_ is the jth volumes dose value, and dDVH_PTV(K)_(D_j_) is the absolute volume (cc) of the jth dose volume in the kth PTV.

CI near 1 indicates that region receiving the reference dose closely matches the shape of the target region (24). IOA have values equal to or greater than 1 and value farther from 1 indicates greater dissimilarity (23).

### Data statistics

Statistical analysis was performed using SPSS Statistics Version 22.0 (IBMs, USA). Analysis of variance was used to evaluate the differences among the three plans. *F* value is used to evaluate the difference between groups. The larger the *F* is, the more significant the equation is, and the better the fitting degree is. The least significant difference was used in pairwise comparisons. All data are expressed as mean ± standard deviation $(\bar{x}\pm \text{S)}$ , and *P*<0.05 indicates a significant difference.

## Results

### Target volume and subvolume comparison

**Figure 3:**
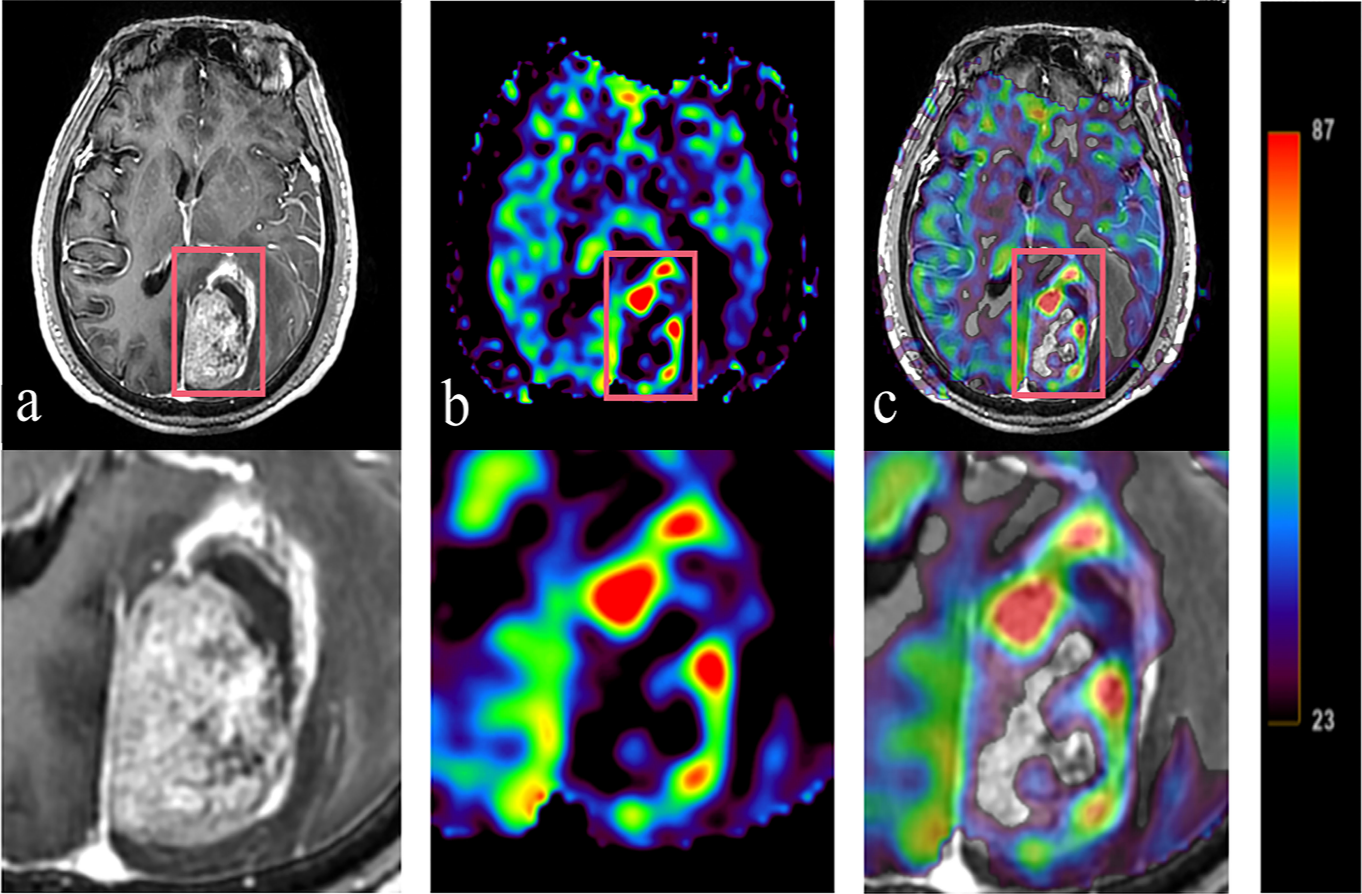
Tumor information is expressed T1WI+C and 3D-ASL images. A 61 year old BMs patient with primary colon carcinoma. According to the enhanced area shown in the T1WI+C images, 3D-ASL showed the uneven distribution of CBF in tumor. The hyperperfused subvolumes are mainly located on the left side of the enhanced edge, while the GTV_H_ and the enhanced area overlap in a large region. Moreover, the fusion image c shows this result better. **(A)** T1WI+C images; **(B)** 3D-ASL images; **(C)** 3D-ASL images fuse with T1WI+C images.

GTV had a volume range of 8.4-118.0 cm^3^, with an average volume of 34.5 cm^3^. And mean GTV_H_ was 17.0 (4.5-58.3) cm^3,^accounting for 49.3% of the GTV. The PTV, PTV_H_ and PTV_N_ were 72.0 cm^3^, 41.5 cm^3^ and 30.5 cm^3^, respectively. The ratios of PTV_H_ to PTV and PTV_N_ to PTV were 57.6% and 42.4%, respectively, as shown in **Table 1** and **Figure 3**.

**Table 1:** Volume and volume ratio comparison.

Target volume	Volume (cm^3^)	Volume ratio (%)
GTV	34.5 ± 21.7	—
GTV_H_	17.0 ± 11.9	49.3
PTV	72.0 ± 34.7	—
PTV_H_	41.5 ± 22.0	57.6
PTV_N_	30.5 ± 18.6	42.4

### Target coverage, CI and IOA comparisons among three plans

As shown in **Table 2**, both conventional and dose painting plans achieved 98% target coverage, even though Plan 2 achieved coverage of PTV_H_ up to 99.90%. Compared with Plan 2, Plan 3 significantly increased the CI levels of PTV_H_ by 12.50% (*P*< 0.05). Owing to the use of targeted gradient doses instead of a group-based uniform dose, CI values of the PTV and PTV_N_ were lower in the dose painting plans than in the conventional plans. For PTV_H_, the IOA values of the three plans were between 1.01 and 1.03 (*P<* 0.05). Meanwhile, the IOA values of PTV and PTV_N_ were between 1.01 and 1.10 (*P<* 0.05).

**Table 2:** Comparison of target coverage, CI and IOA among three plans.

Program		Plan1	Plan2	Plan3	*F*	*P*	*P_1-2_*	*P_1-3_*	*P_2-3_*
Target coverage	PTV	99.60 ± 0.60	99.95 ± 0.13	99.86 ± 0.26	10.995	≤0.05	≤0.05	≤0.05	>0.05
	PTV_H_	99.10 ± 5.19	99.90 ± 0.18	98.40 ± 1.47	2.895	>0.05	>0.05	>0.05	≤0.05
	PTV_N_	99.15 ± 1.41	99.88 ± 0.34	99.76 ± 0.40	10.346	≤0.05	≤0.05	≤0.05	>0.05
CI	PTV	0.75 ± 0.07	0.54 ± 0.07	0.55 ± 0.07	144.791	≤0.05	≤0.05	≤0.05	>0.05
	PTV_H_	0.44 ± 0.10	0.64 ± 0.14	0.72 ± 0.11	78.818	≤0.05	≤0.05	≤0.05	≤0.05
	PTV_N_	0.28 ± 0.09	0.21 ± 0.07	0.21 ± 0.07	12.457	≤0.05	≤0.05	≤0.05	>0.05
IOA	PTV	1.02 ± 0.01	1.10 ± 0.03	1.09 ± 0.04	112.623	≤0.05	≤0.05	≤0.05	≤0.05
	PTV_H_	1.03 ± 0.01	1.02 ± 0.01	1.01 ± 0.02	95.152	≤0.05	≤0.05	≤0.05	≤0.05
	PTV_N_	1.01 ± 0.02	1.08 ± 0.03	1.06 ± 0.03	66.247	≤0.05	≤0.05	≤0.05	≤0.05

F and P were the results of analysis of variance; P_1-2_, Plan 1 vs Plan 2; P_1-3_, Plan 1 vs Plan 3; P_2-3_, Plan 2 vs Plan 3.

### Comparison of the dosimetric indices among the three plans

**Figure 4:**
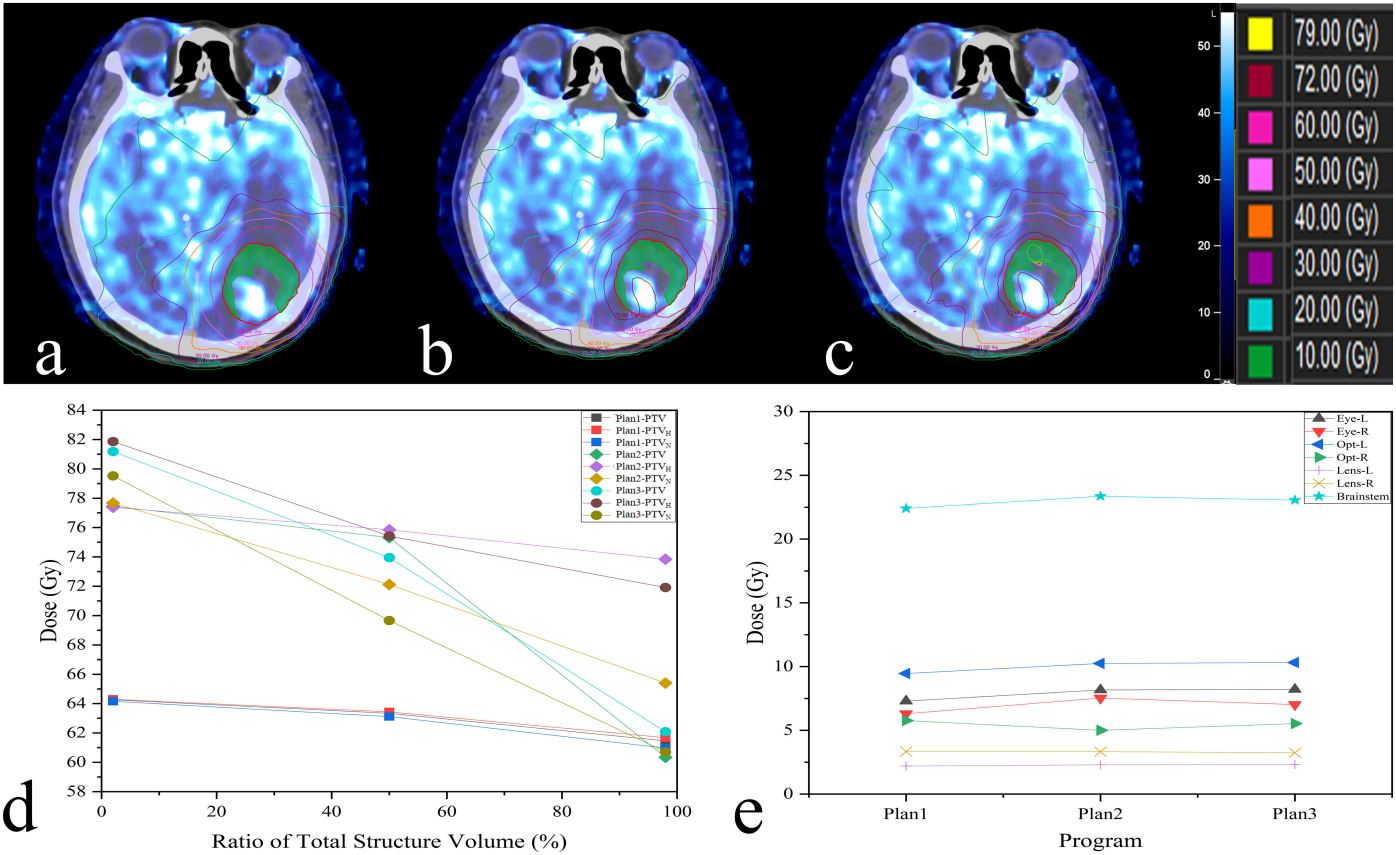
The difference among three plans is shown in this example (**A**:Plan1; **B**:Plan2; **C**:Plan3);. Display on axial slices for dose painting plan showing prescribed 72 Gy around PTV_H_ (green). The dosimetric indices D_2%,_ D_98%_, Dmean and the dose received by OARs are also revealed in **(D, E)**.

The differences in all dosimetric indices of PTVs except D_2%_ were statistically significant (*P*< 0.05). Compared to those of Plan 1, the D_2%_, D_98%_ and D_mean_ of the PTV were increased by 20.18%, 8.34% and 18.38% in Plan2 and by 24.05%, 6.77% and 17.00%, respectively, in Plan 3. The dosimetric indices above for PTV_H_ were increased by 20.50%, 19.32% and 19.60% in Plan 2, while those of Plan 3 were increased by 24.88%, 17.22% and 19.22%. Similarly, the dosimetric indices of PTV_N_ were increased in Plan 2 and Plan 3 (18.81%, 7.17%, 14.31%; 19.69%, 5.15%, 11.80%). Additionally, the D_2%_ values of PTVs showed increasing trends among three plans, while the increment rates of D_98%_ and D_mean_ for Plan 2 were higher than those for Plan 3, as shown in **Table 3** and **Figure 4**.

**Table 3:** Comparison of D_2%_、D_98%_ and D_mean_ among three plans.

Program		Plan1	Plan2	Plan3	*F*	*P*	*P_1-2_*	*P_1-3_*	*P_2-3_*
PTV	D_2%_(Gy)	64.42 ± 0.37	77.42 ± 0.62	79.91 ± 2.28	1818.024	≤0.05	≤0.05	≤0.05	≤0.05
	D_98%_(Gy)	61.17 ± 0.66	66.27 ± 2.07	65.31 ± 2.25	112.920	≤0.05	≤0.05	≤0.05	≤0.05
	D_mean_(Gy)	63.29 ± 0.19	74.92 ± 0.68	74.05 ± 0.84	5178.923	≤0.05	≤0.05	≤0.05	≤0.05
PTV_H_	D_2%_(Gy)	64.38 ± 0.61	77.58 ± 0.52	80.40 ± 2.39	1727.402	≤0.05	≤0.05	≤0.05	≤0.05
	D_98%_(Gy)	61.74 ± 0.69	73.67 ± 0.34	72.37 ± 0.61	6660.500	≤0.05	≤0.05	≤0.05	≤0.05
	D_mean_(Gy)	63.43 ± 0.19	75.86 ± 0.08	75.62 ± 0.29	58745.045	≤0.05	≤0.05	≤0.05	≤0.05
PTV_N_	D_2%_(Gy)	64.39 ± 0.50	76.50 ± 1.19	77.07 ± 2.61	904.842	≤0.05	≤0.05	≤0.05	>0.05
	D_98%_(Gy)	60.55 ± 1.54	64.89 ± 1.83	63.67 ± 1.94	79.330	≤0.05	≤0.05	≤0.05	≤0.05
	D_mean_(Gy)	63.05 ± 0.27	72.07 ± 0.71	70.49 ± 0.88	2551.806	≤0.05	≤0.05	≤0.05	≤0.05

F and P were the results of analysis of variance; P_1-2_, Plan1 vs Plan2; P_1-3_, Plan 1 vs Plan 3; P_2-3_, Plan 2 vs Plan 3.

There was no signifificant difference in D_max_ received by OARs between conventional and dose painting plans (*P* > 0.05). Nevertheless, the dose painting plans slightly increased the D_max_ of all OARs compared to Plan 1, while right eye increased by 5.61% to 0.50 Gy in Plan 3. The other OARs increased by less than 5.40% (2.19% - 5.30%), as shown in **Table 4**.

**Table 4:** Comparison of organs at risk among three plans.

Program	Eye-L(Dmax-Gy)	Eye-R(Dmax-Gy)	Optic nerve-L(Dmax-Gy)	Optic nerve-R(Dmax-Gy)	Lens-L(Dmax-Gy)	Lens-R(Dmax-Gy)	Brainstem(Dmax-Gy)
Plan1	8.74 ± 9.06	8.92 ± 9.99	8.36 ± 12.02	7.36 ± 9.39	2.31 ± 1.54	2.07 ± 1.54	18.68 ± 12.97
Plan2	8.97 ± 8.97	9.39 ± 10.19	8.62 ± 12.21	7.66 ± 9.77	2.37 ± 1.59	2.14 ± 1.55	19.09 ± 13.23
Plan3	8.98 ± 8.75	9.42 ± 10.15	8.67 ± 12.29	7.75 ± 9.83	2.40 ± 1.68	2.15 ± 1.53	19.14 ± 13.30
*F*	0.012	0.039	0.009	0.023	0.038	0.049	0.018
*P*	>0.05	>0.05	>0.05	>0.05	>0.05	>0.05	>0.05
%_(1-2)_	2.63	5.27	3.11	4.08	2.60	3.38	2.19
%_(1-3)_	2.75	5.61	3.71	5.30	3.90	3.86	2.46
%_(2-3)_	0.11	0.32	0.58	1.17	1.27	0.47	0.26
*P_1-2_*	>0.05	>0.05	>0.05	>0.05	>0.05	>0.05	>0.05
*P_1-3_*	>0.05	>0.05	>0.05	>0.05	>0.05	>0.05	>0.05
*P_2-3_*	>0.05	>0.05	>0.05	>0.05	>0.05	>0.05	>0.05

F and P were the results of analysis of variance; %_(1-2)_, % increment between Plan 1 and Plan 2; %_(1-3)_, % increment between Plan 1 and Plan 3; %_(2-3)_, % increment between Plan 2 and Plan 3; P_1-2_, Plan 1 vs Plan 2; P_1-3_, Plan 1 vs Plan 3; P_2-3_, Plan 2 vs Plan 3.

## Discussion

This study demonstrated that BMs can be divided into high and low perfusion subvolumes based on 3D-ASL. We therefore presented a new method for assessing the clinical feasibility of dose painting plans directed by 3D-ASL for BMs. The results showed that the technique above can be completed feasibly without increasing the dose delivered to OARs which holds the potential to achieve better control of BMs than traditional RT treatment.

BMs are significant health problems whose incidence is increasing, and the median survival time is only 1-2 months without treatment (1, 25). Numerous studies have indicated that increasing the radiation dose significantly improves the local control of BMs, and some patients have achieved long-term survival (26, 27). Therefore, RT is an irreplaceable treatment for BMs. However, increasing radiation dose, particularly to vascular endothelial cells and glial cells, is associated with elevated toxicity and reduced tolerance to treatment (28). Thus, dose escalation without guidance is relevant to high risk of radiation induced brain injury.

Some studies have shown that BMs are highly heterogeneous according to their pathological sources or even sites of pathological origin (29). Brown et al. proposed that the blood flow in hypoxic tissue is slower than that in normal tissue and cannot satisfy the oxygen requirement of rapidly proliferating tumor cells because of highly irregular tumor vessels, arteriovenous shunts, blind ends, an incomplete basement membrane of vascular epithelial cells and other factors (30). Due to the uneven distribution of blood flow and cancer cells, BMs appear as radiation-sensitive regions with high perfusion, hypoxic radiation-resistant regions with low perfusion, and necrotic regions. Furthermore, the isoeffective dose can be up to three times higher under hypoxic conditions than under normoxic conditions (31). However, the group-based uniform dose given under a conventional plan cannot guarantee a sufficient dose for radiation-resistant regions, which eventually leads to further tumor progression and recurrence; local control failures are not uncommon under conventional treatment.

Dose escalation according to heterogeneity is essential, and primary task is to determine the tumor target subvolume with biological images. Perfusion-weighted MR can be used for quantitative analysis of blood flow parameters (9, 11, 16, 32). The noninvasive technology 3D-ASL reflects angiogenesis and other functional features of tumor microvascular system, rather than reflecting only morphology as CT and conventional MR (9). The amount of blood in one slice was evaluated by measuring the signal reduction after arterial blood saturation with radio frequency pulse. Therfore, ASL observe cerebral perfusion without using contrast agent, and has good safety and repeatability. Yukie et al. demonstrated that the area under the curve value of ASL for the recognition of hypoxic areas reached 0.83 based on the hypoxic tracer ^18^F-fluoromisonidazole (10). Our previous studies also demonstrated that the CBF variations in brain tissue and BMs following radiation dose gradients could be quantified by 3D-ASL. In this paper, 49.3% of the GTV was within a region of low CBF which showed that subvolume segmentation based on CBF map using 3D-ASL is feasible (33).

A major concern regarding the implementation of dose escalation for specific subvolumes can be solved by dose painting which aims to improve tumor control without adding doses to OARs (34). Dose painting guided by positron emission computed tomography (PET) has been widely studied in head and neck tumors, but the application of PET is indisputably limited at present because of its invasiveness and high price (35, 36). Perfusion technique has been proved to be used to improve radiotherapy regimens and provide more biological information (37).Thus, in the present study, dose painting was used to achieve dose escalation in hypoperfused subvolumes that were recognized and segmented by 3D-ASL. The results demonstrated that compered those of Plan 1, the D_2%_, D_98%_ and D_mean_ of the PTV_H_ were increased by 20.50%, 19.32% and 19.60% in Plan 2, while those of Plan 3 were increased by 24.88%, 17.22% and 19.22%. Compared to the conventional plans of 60 Gy, the dose painting plans significantly increased the dosimetric indices. However, there were two trends. On the one hand, the maximum dose of PTVs all showed increasing trends among three plans. On the other hand, the constrained dose painting plans had better average and minimum doses than the other two plans, and this was the case for all PTVs. Thorwarth et al. noted that dose of up to 82 Gy may be applied to head and neck tumors without increasing toxicity, but constraints for normal tissue were not stated in their work (38). Our results indicated that the dose delivered to OARs was increased less than 4.10% (2.19%-4.08%) except for the right eye (5.27%; 0.47 Gy) when the prescription dose of PTV_H_ was increased by 20% in Plan2. Undoubtedly, the absolute dose was still far below the dose constraint. In addition, the constrained dose painting plans outperformed the unconstrained plans in terms of OARs protection.

Both conventional and dose painting plans achieved target coverage of more than 98%. Likewise, the constrained dose painting plans had better target coverage than unconstrained and conventional plans. Chang et al. pointed out that reducing the uniformity of radiation dose is beneficial for dose escalation and OAR protection (35). The IOA values of different PTVs in three plans are less than or equal to 1.10, which indicates that good targeted dose distribution can be achieved. Meanwhile, it is necessary to confirm CI because the shape of subvolumes defined by perfusion is often irregular. In this work, CI of the PTV_H_ was effectively guaranteed through the dose painting plans, which further proved the feasibility of our experimental method.

There are still some limitations in this study. First, PLD is one of the important parameters for accurate evaluation of CBF. However, in practice, it is difficult to ensure that PLD is set according to the specific conditions of patients to adapt to the arrival time of labeled arterial blood. Second, the effect of dose painting plans for BMs still needs to be confirmed in clinical practice. Relevant research is under way.

## Conclusions

In summary, CBF maps based on 3D-ASL could be used for guiding subvolumes segmentation. Dose painting guided by different CBF variations offeres a novel approach for BMs RT. Safe dose escalation without additional radiation doses to OARs provides an effective individualized dose painting strategy for BMs patients.

## Data availability statement

The raw data supporting the conclusions of this article will be made available by the authors, without undue reservation.

## Ethics statement

The studies involving human participants were reviewed and approved by Institutional Review Board of the Shandong Cancer Hospital. Written informed consent for participation was not required for this study in accordance with the national legislation and the institutional requirements.

## Author contributions

CH, HY, and YY designed the study and wrote the initial draft of the manuscript. GG contributed to the design of the study and the analysis and interpretation of data and assisted in the preparation of the manuscript. LW, YS, and JL contributed to data collection and interpretation, and critically reviewed the manuscript. All authors contributed to the article and approved the submitted version.

## Funding

This work was supported by Key Support Program of Natural Science Foundation of Shandong Province (Grant No. ZR2019LZL017), the Taishan Scholars Project of Shandong Province (Grant No. ts201712098) and National Key Research and Development Program of China (Grant No. 2017YFC0113202).

## Acknowledgments

We gratefully acknowledge Qingtao Qiu for his support with text proofing.

## Conflict of interest

The authors declare that the research was conducted in the absence of any commercial or financial relationships that could be construed as a potential conflict of interest.

## Publisher’s note

All claims expressed in this article are solely those of the authors and do not necessarily represent those of their affiliated organizations, or those of the publisher, the editors and the reviewers. Any product that may be evaluated in this article, or claim that may be made by its manufacturer, is not guaranteed or endorsed by the publisher.
